# Mr. William Pierce Hagee

**Published:** 1916-04

**Authors:** 


**Mr. William Pierce Hagee,** President of the Katharmon Chemical Co., of St. Louis, died Feb. 3.

